# Pulmonary function after less invasive anterior instrumentation and fusion for idiopathic thoracic scoliosis

**DOI:** 10.1186/1748-7161-8-14

**Published:** 2013-08-21

**Authors:** Geertje C Huitema, Rob C Jansen, Edward Dompeling, Paul Willems, Ilona Punt, Lodewijk W van Rhijn

**Affiliations:** 1 Department of Orthopedic Surgery, Westfriesgasthuis, Hoorn, NH, The Netherlands; 2 Department of Orthopedic Surgery, Maastricht University Medical Centre, Maastricht, The Netherlands; 3 Department of Paediatric Pulmonology, Maastricht University Medical Centre, Maastricht, The Netherlands; 4 Research School CAPHRI, Maastricht University, Maastricht, The Netherlands

**Keywords:** Adolescent idiopathic scoliosis, Pulmonary function tests, Anterior fusion, Anterior instrumentation

## Abstract

**Purpose:**

Standard thoracotomy for anterior instrumentation and fusion of the thoracic spine in idiopathic scoliosis may have detrimental effects on pulmonary function. In this study we describe a less invasive anterior surgical technique and show the pre- and postoperative pulmonary function with a minimum follow-up of 2 years.

**Methods:**

Twenty patients with Lenke type 1 adolescent thoracic idiopathic scoliosis were treated with anterior spinal fusion and instrumentation. The mean preoperative Cobb angle of the thoracic curve was 53° ± 5.8. Pulmonary function tests (PFT) and radiographic evaluation was performed.

**Results:**

The mean postoperative correction in Cobb angle of the thoracic curve was 27° ± 8.2 (49%).

The mean preoperative FEV1 was 2.81 ± 0.43 L, which increased to 3.14 ± 0.50 L at 2 years postoperatively (*P* = 0.000). The mean FEV1% did not change (89.60 ± 7.49% preoperatively, versus 90.53 ± 5.95% at 2 years follow-up, *P* = 0.467). The TLC increased from 4.62 ± 0.62 L preoperatively to 5.17 ± 0.63 L at 2 years follow-up (*P* = 0.000). The FEV1% at two years of follow-up improved to 104% of the FEV1% predicted value. The FEV1 improved to 97% of the FEV1 predicted value.

**Conclusion:**

Anterior spinal fusion for idiopathic scoliosis by means of a minimal open thoracotomy proved to be a safe surgical technique that resulted in an improvement of pulmonary function. Our results are similar to those of thoracoscopic procedures reported in literature.

## Introduction

The primary indication for spinal fusion in the treatment of thoracic scoliosis is the prevention of curve progression by stable arthrodesis. The surgeon should aim for a maximum safe correction of the deformity with restoration of coronal and sagittal balance while fusing as few motion segments as needed. Besides prevention of curve progression, deterioration of pulmonary function should be avoided. In patients with thoracic scoliosis with large curves (greater than 50°) the thoracic cage deformity has a profound effect on respiratory mechanics, which is usually that of restrictive lung disease [1]. Surgical correction of thoracic scoliosis by thoracotomy can compromise pulmonary function as well [2-5].

The main advantage of an anterior approach to the thoracic spine is preservation of mobile segments through a shorter fusion: In some scoliosis’ curve patterns both proximal and distal levels can be saved, which would have been instrumented in a posterior spinal fusion [6-8]. Other advantages of an anterior approach are resection of the growth plate and thereby prevention of the crankshaft phenomenon, and correction of the curve by compression rather than distraction, which has a lower rate of neurological complications [9].

There are three techniques for an anterior approach of the thoracic spine: (A) Fully open by means of a standard thoracotomy; (B) completely thoracoscopic; and (C) minimally open (with or without thoracoscopy). These three techniques seem to have comparable clinical results in scoliosis treatment, but they differ in perioperative problems [10,11]. Whereas the standard thoracotomy technique is major invasive surgery, the thoracoscopic technique is technically demanding and has a significant learning curve. The minimal invasive technique has the advantage of less soft tissue damage with a shorter learning curve and a direct view on the spine.

Several studies have documented effects on pulmonary function testing after thoracotomy as far as 2 to 5 years later [4,5,12]. For posterior fusions typically, pulmonary function has improved at 2 years follow-up, as compared to the baseline level [4,5,13]. In order to prevent deterioration of pulmonary function, less invasive surgical techniques have been developed [10,14,15].

In the present study we used a minimal open thoracotomy for anterior instrumentation and fusion of the thoracic spine in scoliosis treatment. The surgical technique is described as well as the radiological results and the changes in pulmonary function are evaluated.

## Materials and methods

A consecutive series of twenty patients with adolescent thoracic idiopathic scoliosis Lenke type 1 who underwent minimal open anterior spinal fusion in the Maastricht University Medical Center between 2000 and 2007 were included for this study.

All patients were treated by a minimal invasive anterior approach for selective thoracic correction and fusion. The technique is described in detail in the surgical technique section. For instrumentation the Eclipse® system (Medtronic Sofamor Danek) was used with 4.5 mm rods. A minimum follow-up of two years was required. The mean follow-up was 3 years ± 0.95 (range 2–5 years).

Patient characteristics are shown in Table 1. The mean age at the time of operation was 16 ±3.1 years (range 12–23). The pulmonary function tests (PFT) consisted of forced expiratory volume in 1 second (FEV1), the FEV1 percentage of the vital capacity (FEV1%), and the total lung capacity (TLC). The FEV1 and the FEV1% values are critically important in the diagnosis of obstructive and restrictive pulmonary diseases. The forced expiratory volume in one second is a measurement both of volume and of mean flow over the first second. The reduction in forced expiratory volume in one second reflects the total effects of reduction in total lung capacity, obstruction of the airways, loss of lung recoil, and relatively uncommon, gross weakness of respiratory muscles. We chose these parameters because they provide an adequate assessment of the volume and flow functions as measured by the pulmonary function tests [4]. All pulmonary function tests were performed on a computerized spirometer. According to international standards, the highest FEV1 and FVC values of three reproducible curves were used for analysis. It is important to present pulmonary function values as percentage predicted values because in immature patients absolute values of pulmonary function increase by growth. Normalizing these values for age, height and sex corrects largely for the increase in lung volumes because of growth. These corrected values are called percentage predicted values. The percentage predicted values are most appropriate to compare preoperative and postoperative values for a given approach [16].

**Table 1:** Patient characteristics and preoperative and postoperative thoracic Cobb angle and pulmonary function tests

					**Preoperative**				**Postoperative**			
**Patient no.**	**Age**	**Sex**	**Length**	**Number of fused levels**	**Cobb Th**	**FEV1**	**FEV1%VC**	**TLC**	**Cobb Th**	**FEV1**	**FEV1%VC**	**TLC**
1	17	F	168	7	50	3.13	97.4	4.94	15	3.45	84.35	5.49
2	19	F	165,5	7	53	2.45	78.6	5.3	20	2.54	78.84	5.3
3	22	F	162,5	7	49	2.32	78.8	4.97	31	3.12	89.8	5.21
4	16	F	169,5	5	55	2.83	85.7	3.83	15	3.19	90.37	5.56
5	14	F	161	5	44	2.92	84.5	4.03	31	3.18	89.7	4.95
6	15	M	172	6	63	3.61	91.80	5.53	51	4.35	85.96	6.98
7	22	F	165	6	47	3.27	95.3	4.95	25	3.07	96.60	5.03
8	16	F	157	7	64	2.87	82.3	4.10	27	3.13	89.11	4.84
9	14	F	158	5	53	2.75	91.1	4.29	22	3.17	95.4	4.90
10	18	F	171	5	50	3.33	96.21	5.25	26	3.61	97.29	5.36
11	14	F	153	6	56	2.76	93.2	4.01	24	3.02	95.1	4.64
12	14	F	179	7	54	2.54	75.2	5.89	24	2.66	76.77	6.22
13	23	M	167	4	50	3.23	96.00	4.56	31	3.89	97.01	4.98
14	14	F	149,5	5	56	1.73	87.2	3.75	34	2.06	92.18	4.01
15	16	F	166	6	49	3.08	86.6	4.94	27	3.25	87.63	5.24
16	12	F	153	6	63	2.18	86.90	3.88	41	2.44	86.20	4.50
17	15	F	168,5	6	56	2.86	92.40	4.86	31	3.33	96.3	5.25
18	17	F	166,5	5	52	2.85	95.90	4.71	25	3.37	96.40	5.23
19	14	F	161	6	48	2.83	91.12	4.50	25	3.06	90.90	5.23
20	15	F	154,5	6	44	2.64	105.60	4.01	24	2.94	94.60	4.51

Age in years.

Sex *F* female, *M* male.

Length in centimeters.

Cobb Th Cobb angle of main thoracic curve in degrees.

FEV1 in liter (L).

FEV1%VC in %.

TLC in liter (L).

Statistical analysis was performed using SPSS (version 17.0, SPSS Inc, Chicago). Pearson correlation analysis was used to identify linear relations between variables. To compare differences between preoperative and postoperative pulmonary function tests the paired t-test was used. All probability values (*p*) were calculated within a confidence interval of 95%.

### Surgical technique of the minimal invasive anterior approach

**Figure 1:**
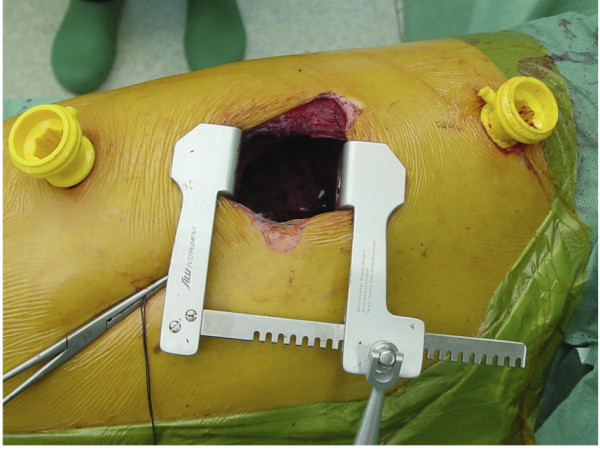
Thoracotomy and portal placement.

After general anesthesia with double lumen intubation for single lung ventilation the patient was positioned in lateral decubitus position. The thoracotomy (length 7 cm) on the convex side was placed over the apex of the scoliosis as determined by fluoroscopy. After making a skin incision, the latissimus dorsi muscle was spared and retracted posterior with a blunt retractor. The serratus anterior muscle was dissected in the direction of its fibers. The rib was dissected subperiostally over a length of 10 to 12 cm and isolated from its periosteal bed. The piece of rib is removed and preserved in a wet gauze for bone grafting. Osteotomies of the resected rib were performed at the anterior and posterior borders of that rib. At that time point, single lung ventilation was started with controlled collapse of the left lung. If necessary the lung could be retracted with a (endoscopic) lung retractor. Generally two extra incisions (length 2 cm) for the portals were needed (Figure 1). The placement of the portals is critical since the vertebrae at these levels are at the greatest angle in relation to the apex of the curve. The technique for the placement of the portals is similar as described for thoracoscopic procedures [17,18]. After screw instrumentation complete discectomies are performed and the disc space is filled with impacted morsellised bone. Later in this series we also used Tri-Calcium-Phosphate (Biosorb®, SBM, Lourdes) to fill the disc space. The ribs and muscle layers are closed on a standard way and the skin incisions are closed with intra-cutaneous sutures.

Fluoroscopy is used to insert all the screws.

### Postoperative regimen

A chest tube is placed in all patients. Patients wear a brace for 3 months.

## Results

**Figure 2:**
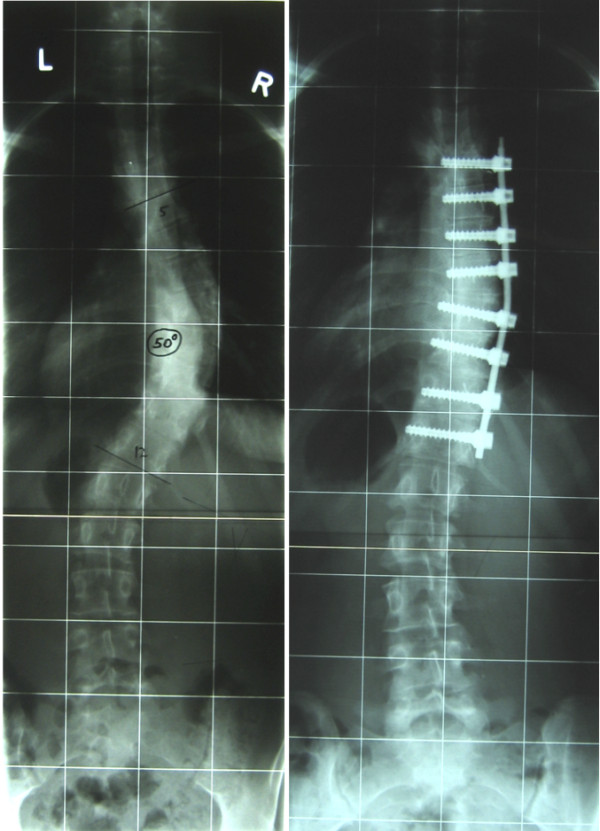
Preoperative curve of 50° on AP radiograph (left) and postoperative curve of 20° (right).

**Figure 3:**
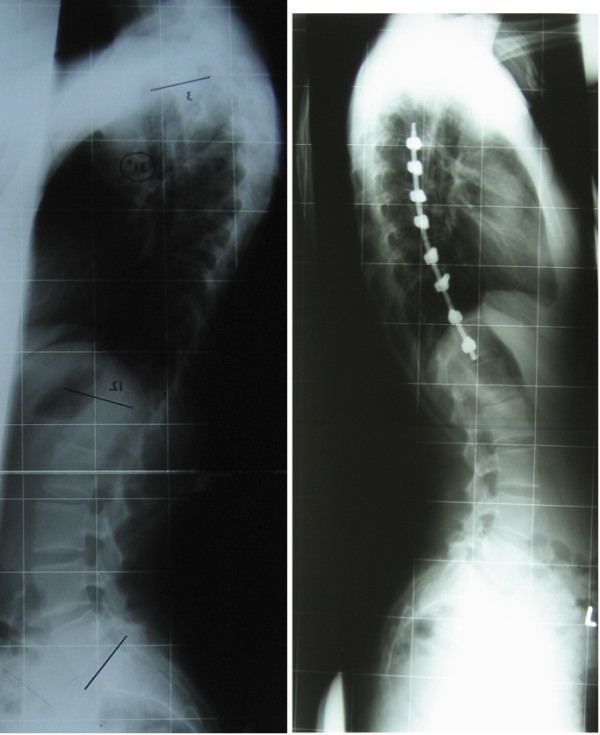
Preoperative curve of 69° on lateral radiograph (left) and postoperative curve on the right.

Table 2 shows patient characteristics and absolute pulmonary function test results. In Figures 2 and 3 preoperative and postoperative radiographs are shown. The mean preoperative Cobb angle of the main thoracic curve was 53° ± 5.8 (range 44-64°). Postoperatively, the mean Cobb angle of the main (instrumented) thoracic curve after a minimum follow-up of 2 years was 27° ± 8.2 (range 15-51°). The mean correction percentage of the instrumented thoracic curve was 49%. The mean correction on bending radiographs of the main thoracic curve was 44% (range 10-62%). The mean number of fused levels was 6. In 13 of the 20 patients the fused levels Th5-Th12 and Th6-Th12. The average blood loss was 600 cc. The average time of surgery was 5.10 hours.

**Table 2:** Mean preoperative and postoperative absolute and predicted pulmonary function test parameters

	**FEV1**	**FEV1%VC**	**TLC**	**Predicted FEV1**	**Predicted FEV1%VC**	**% predicted FEV1**
Preoperative	2,81	89,6	4,62	3,24	87,2	86,7
Postoperative	3,14	90,53	5,17	3,27	87	96,0
*p*	0.000	0.467	0.000			

FEV1 in liter (L).

FEV1%VC in %.

TLC in liter (L).

Predicted predicted values.

*P value* using the paired samples t-test.

Table 2 shows the mean preoperative and postoperative absolute and predicted pulmonary function test parameters. The mean preoperative FEV1 was 2.81 L that increased to 3.14 L at 2 years follow-up (*P* = 0.000). The FEV1% was 89.60% preoperatively and 90.53% at 2 years follow-up (*P* = 0.467). The TLC increased from 4.62 L preoperatively to 5.17 L at 2 years follow-up (*P* = 0.000). The predicted FEV1 and the predicted FEV1% did not improve (Table 2). The % predicted FEV1 however did improve: 86,7% preoperative versus 96% postoperative. No statistical significant correlation was found between pre- and postoperative sagittal alignment (sagittal Cobb angles of Th5-Th12, Th10-Th12, Th12-L1 and L1-L5) and pre- and postoperative pulmonary function.

No vascular, neurological or pulmonary complications were observed. None of the minimally invasive anterior procedures needed conversion into a large standard thoracotomy.

In one of the patients a pseudarthrosis and breakage of the rod occurred. The anterior instrumentation was removed and an instrumented posterior spinal fusion was performed.

Overall, the FEV1 improved in 19 of 20 patients, TLC improved in 19 of 20 patients and FEV1% improved in 15 of 20 patients. In 6 patients no improvement was seen postoperatively of either FEV1 or FEV1% or TLC.

## Discussion

In this study we analyzed pulmonary function after a minimal invasive anterior approach for spinal correction and fusion of adolescent idiopathic thoracic scoliosis. The results demonstrate a significant improvement of FEV1, FEV1% and TLC after a minimal invasive thoracotomy at a minimum of two years of follow-up. Our results are comparable to those of other studies that showed an improvement of absolute PFT results between 12% and 15% after thoracoscopic procedures [3,19].

In immature patients the absolute values of pulmonary function increase by growth. Normalizing pulmonary function values for age, height and sex corrects largely for the increase in lung volumes because of growth. These corrected values are called percentage predicted values. The percentage predicted values are most appropriate to compare preoperative and postoperative values for a given approach [16]. The improvement of % predicted FEV1 (86,7% preoperative versus 96% postoperative) therefore is not the effect of age but a true improvement.

The anterior approach to the thoracic spine by standard thoracotomy has been reported to have long-term detrimental effects on pulmonary function. Graham et al. [5] showed that pulmonary function test values after anterior spinal fusion via rib resection thoracotomy returned to within 94% to 96% of baseline by the 2-year follow-up visit, but were still statistically less than the preoperative values (*P* ≤0.05). Sudo et al. [20] showed that the average percent-predicted forced vital capacity and forced expiratory volume in 1 second were significantly decreased during long-term follow-up (12–18 years, average 15,2 years) measurements (73% and 69%; P = 0.0004 and 0.0016, respectively).

The thoracoscopic approach to the spine has been suggested to be less invasive and thereby could have a less detrimental effect on pulmonary function. There are few studies that compare the effect of surgical approach to the thoracic spine on pulmonary function. Lenke et al*.*[3] compared open versus endoscopic anterior fusion combined with posterior fusion and found no significant difference in pulmonary function after 2 years of follow-up. Lonner et al*.*[19] showed in a study comparing thoracotomy, thoracoscopy and a thoracoabdominal approach better pulmonary function test results after thoracoscopy compared with thoracotomy.

The size of the thoracotomy appears to be of influence on postoperative pulmonary function. This has been shown in a study by Namboothiri et al*.*[21]. They compared two groups with adolescent idiopathic thoracic scoliosis who underwent two-stage surgery using an anterior release and posterior fusion and instrumentation. One group had a standard large thoracotomy in which the thoracotomy size was 15–20 cm and another group had a minimal open thoracotomy in which the thoracotomy size was 5–7.5 cm. Both groups showed a decline in FEV1 and FVC values at 2 weeks and 3 months after the thoracotomy, but this decline was smaller in the small thoracotomy group. In our study the length of the skin incision was 7 cm. The rib dissection subperiostally was 10–12 cm length and we used 2 extra portals of 2 cm length each. This makes our incision length 14–16 cm, but the size of the thoracotomy that is of influence on pulmonary function is 10–12 cm. This is between a large thoracotomy size and mini open. Our patients demonstrated a significant improvement of FEV1, FEV1% and TLC. We performed anterior instrumentation and fusion together with an anterior release, which requires single lung ventilation. Single lung ventilation can cause high airway pressures and ventilation-perfusion mismatches which can lead to life threatening complications such as air throughout the chest, mediastinum, abdomen, and subcutaneous tissues [22]. However, we did not have such life threatening complications in our series of patients.

Newton et al. [23] showed that preoperative pulmonary function test values were the strongest predictors of 2-year pulmonary function after surgery. They also showed that, two years after anterior thoracic scoliosis correction, the thoracoscopic group was superior with regard to both absolute pulmonary function test volumes and percentage predicted values as compared with the open thoracotomy group that included a thoracoplasty.

The results of our study demonstrate improved pulmonary function test results after minimal invasive thoracotomy.

We used fluoroscopy to insert all the screws. One could state that our technique leads to more fluoroscopy use but this is comparable to the use of fluoroscopy with the screw placement in posterior instrumentation.

## Conclusion

In adolescent idiopathic scoliosis patients the less invasive anterior approach for correction and fusion of the thoracic spine appeared to be a safe surgical technique without detrimental effects on pulmonary function.

## Competing interests

The authors declare that they have no competing interests.

## Authors’ contribution

GCH carried out the acquisition of data, the analysis and interpretation of data and drafting of the manuscript. RCJ carried out the acquisition of data and drafting of the manuscript. ED performed pulmonary function test and revised the manuscript. IP carried out the analysis and interpretation of data and revised the manuscript. PW carried out the analysis and interpretation of data and revised the manuscript. JWR carried out the analysis and interpretation of data and revised the manuscript. All authors read and approved the final manuscript.
